# Small Bowel Perforation Due to Blunt Trauma of Left Leg With an Incarcerated Inguinal Hernia: A Case Report

**DOI:** 10.3389/fsurg.2021.710417

**Published:** 2021-09-27

**Authors:** Jianli Shao, Long Sun, Qinghui Fu

**Affiliations:** ^1^Traditional Chinese Medical Hospital of Sanmen, Sanmen, China; ^2^School of Medicine, First Affiliated Hospital, Zhejiang University, Hangzhou, China

**Keywords:** case report, hernia, small bowel perforation, surgery, misdiagnosis

## Abstract

We report a rare case of a 77-year-old man with a known left inguinal hernia presenting with groin pain following a blunt trauma of the left leg. Diagnosis of small bowel perforation away from the hernia was obtained only in surgery. Difficulty in preoperative diagnosis, rarity of histologic pattern, and surgical challenges make this case very interesting for surgeons and radiologists. Our conclusion upon dealing with the situation is that the diagnosis of small bowel perforation following blunt injury to a non-abdominal trauma is rare and difficult. The association between inguinal hernia and non-abdominal trauma may result in small bowel injuries that normally do not appear. Therefore, clinicians should be cautious in treating non-abdominal trauma patients with inguinal hernias.

## Introduction

Inguinal hernias are the most commonly presented abdominal hernias, with approximately 20 million people operated on annually throughout the world (1). The diagnosis of inguinal hernias can be done easily by a clinical exam. But bowel perforation away from the herniated bowel caused by an indirect blunt trauma is rare and can have important consequences. It is more difficult to diagnose. This is especially true when the lesion is located outside the abdomen, as the perforation of the small intestine is less common. Failing to diagnose and treat these injuries meaningfully increases the morbidity and mortality. As the case described below reveals, the presence of inguinal hernia is a major factor that can also lead to intestinal perforation, regardless of whether the force is applied inside or outside of the abdomen.

## Case Description

A 77-year-old male, with a long standing reducible left inguinal hernia, presented to the emergency department of our hospital with a 4 h history of left groin mass pain and incarceration following a fall while walking. The mass was about the size of a “fist”, accompanied by persistent pain that could not be relieved. In the emergency department the patient was hemodynamically stable with blood pressure of 110/78 mmHg and heart rate of 85 beats per minute. The patient did not have a history of co-morbidities or abdominal operations. The abdomen was flat and soft, and no obvious traumatic changes were observed in the abdominal wall or groin skin. Bruising was observed at the root of the left thigh, and skin abrasions were observed at the trochanter of the left femur. At the local examination the patient was shown to have a discrete painful abdomen with the maximum of intensity in the lower abdomen. There was a mass of about 10^*^8 cm in the left groin. It could not be reabsorbed. The mass was negative in light transmission test and positive in tenderness.

**Figure 1:**
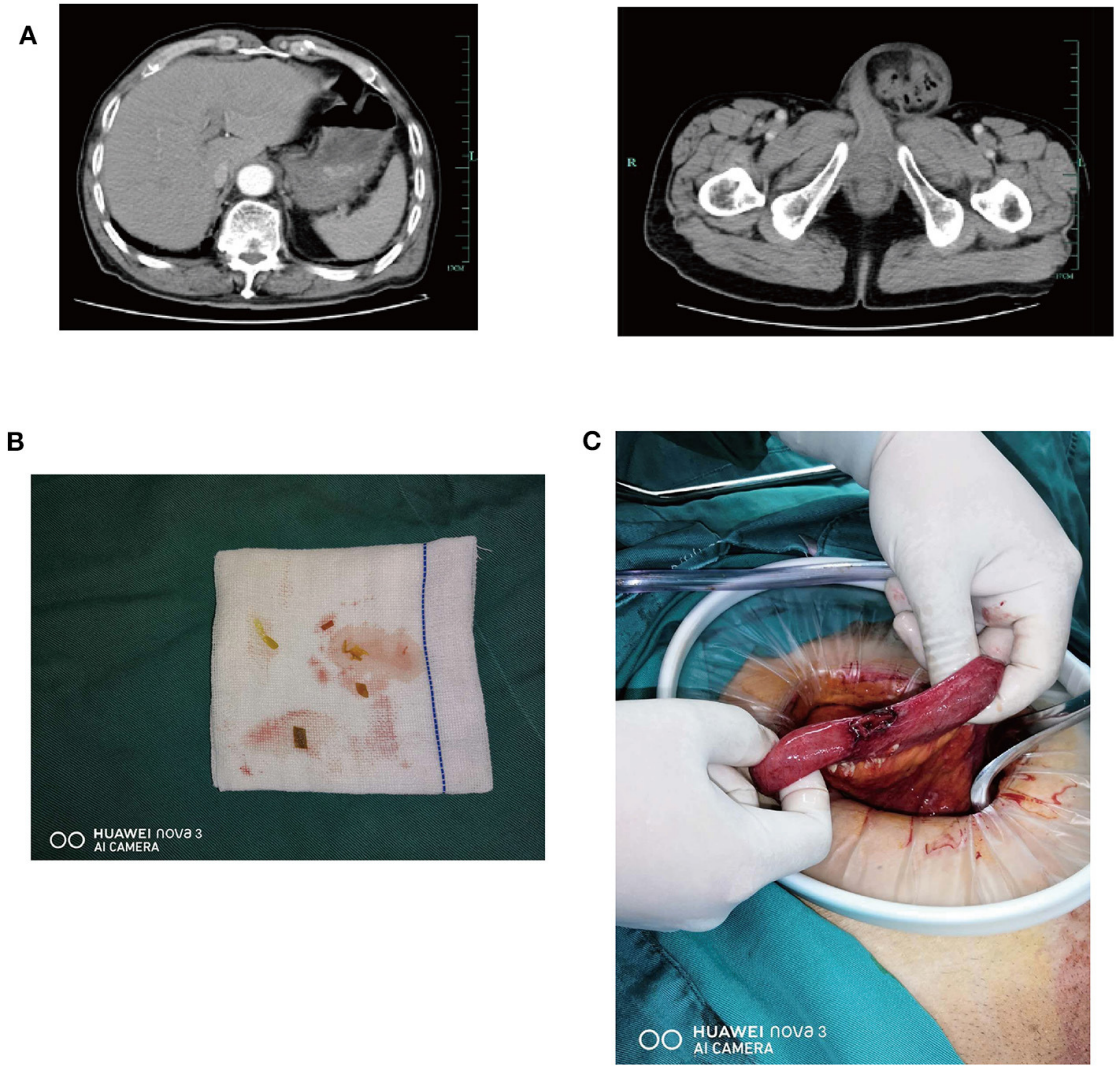
**(A)** Contrast enhanced CT of the abdomen in a 77-year-old male with a non-abdomen trauma. In the left CT photograph of the upper abdomen, no free abdominal gas has been found. In the right CT photograph of the pelvis, the hernia contents of the left incarcerated inguinal hernia can be seen clearly. **(B)** A small amount of food in ascites can be seen in the left groin which suggests the possibility of perforation of the intraperitoneal intestine. **(C)** In the middle part of the small intestine, there was a laceration of about 1.0 cm with obvious adhesion of purulent moss.

We initially performed laboratory tests to reveal white blood cell count (3.2*10∧9/L), the neutrophil ratio (80.9%), and Lymphocyte ratio (15.6%). The remaining blood tests were normal. The abdominal X-ray in standing position did not show the presence of free intraperitoneal air. The upper X-ray did not show the presence of fracture and dislocation. Abdominal enhanced CT- scan (Figure 1A) showed a left inguinal hernia, a small stone in the right kidney, and a small amount of effusion in the pelvic cavity. A preoperative diagnosis of incarcerated inguinal hernia (left), inguinal hernia (right), and hypertension was made. Because of the incarcerated inguinal hernia, emergency surgery was performed. A parallel incision was made in the left inguinal ligament, and the intraoperative local tissue edema was obvious. When the hernia sac was opened, part of the sigmoid colon and intestine were herniated and incarcerated, and the color and activity of the intestine were normal. The hernia contents were loosened and re-incorporated, and turbidities were observed in the ascites. Careful examination revealed a small amount of food residue overflow, which was considered to be from intraperitoneal intestinal perforation (Figure 1B). The hernia contents were restored, the distal excess hernia sac was removed, the proximal high ligation was performed, and the inguinal incision was temporarily filled with damp gauze. A midumbilical incision was made in the middle and lower abdomens. After entering the abdomen, extensive exudative changes were observed in the abdominal cavity, with multiple pus moss and food residue visible. A rupture of about 1.0 cm was observed in the middle part of the small intestine, with obvious adhesion of pus moss around the abdomen (Figure 1C). After repairing the ruptured hole, no ruptured perforation was found in other intestinal tubes. A postoperative diagnosis of small bowel perforation, incarcerated inguinal hernia (left), inguinal hernia (right), and hypertension was made. His postoperative recovery was unremarkable and he was discharged from hospital after a few days; subsequent histology showed no obvious cause to suspect a perforation.

This patient was preoperatively considered for an incarcerated inguinal hernia (left side) and an inguinal hernia (right side). Considering that it was an emergency surgery at that time, in principle, only an incarcerated hernia could be treated. Moreover, the actual surgery confirmed small bowel perforation, so the surgical incision was a contaminated incision. Hernia repair could only be performed with the most basic high ligation of the hernia sac, which had the problem of higher risk of recurrence after surgery than elective tension-free repair. Therefore, only high ligation of the hernia sac was performed for the left-sided hernia, and the right-sided inguinal hernia was left for later elective surgery.

## Discussion

Groin hernias are caused by a defect of the abdominal wall in the groin area and are comprised of inguinal and femoral hernias. Inguinal hernias are more common in men (2). Incarceration and strangulation of the intestinal structures are the most frequently reported complications of unrepaired inguinal hernia (3). Generally speaking, the possibility of abdominal organ injury always occurs when the external force acts on the abdomen. Although peritonitis may result from rupture and perforation of the small intestine due to indirect violence, such injury is usually located in a relatively fixed part of the small intestine within the abdominal cavity. In a previous study, it was found that intestinal perforation followed blunt abdominal trauma with pre-existing inguinal hernia (4, 5). In this case, the force was neither placed on the abdomen, nor on a relatively fixed segment of the small intestine. The absence of typical abdominal symptoms at the time of the emergency room can mislead physicians and lead to delayed diagnosis. At the same time, the patient also had an incarcerated hernia in the left groin, which was easy to interfere and misjudge the clinical diagnosis.

Intraluminal pressure can be increased by the increase of intra-abdominal pressure, and intestinal loops overlying the hernia aperture can blow out over the aperture (6). In 1995, Reynolds explained that additional pressure applied to the sealed loop generated enough intraluminal pressure to cause a perforation (7). This direct trauma to an inguinal hernia can give spikes of pressure >300 mmHg, when only 150–260 mmHg is enough to induce an intestinal loops rupture. Therefore, the following may be the cause of intestinal rupture. When part of the small intestine was herniated, a segment of the intestine in the abdominal cavity was strained. The momentary change of posture increased abdominal pressure, resulting in momentary changes in the position of the small intestine. And the powerful external force tore the small intestinal wall or damaged the intestinal wall, and then perforation occurred. The injured small intestine will return to the abdominal cavity due to its most mobile segment and its own peristalsis, and the leaked contents of the small intestine will also enter the abdominal cavity. Inflammatory stimulation led to increased intraabdominal pressure, which in turn led to partial sigmoid intestinal herniation and incarcerated hernia.

In our case report the fact that the patient had abdominal pain without abdominal muscle tension and peritonitis irritation sighs made the diagnosis difficult. It was found after an accidental fall and trauma. CT examination did not reveal the possibility of free air in the abdomen. Therefore, for patients with abdominal pain and incarcerated hernia, it is important to ask whether the abdominal pain or incarcerated hernia came first in order to identify whether the incarcerated hernia caused the perforation or the perforation caused the incarcerated hernia.

## Conclusion

The diagnosis of small bowel perforation following blunt injury to a non-abdominal trauma is rare. The association between inguinal hernia and non-abdominal trauma may result in injuries that normally do not appear. Therefore, clinicians should be cautious in treating non-abdominal trauma patients with inguinal hernias. A high degree of suspicion can give rise to early diagnosis and treatment which offer good outcomes.

## Data Availability Statement

The original contributions presented in the study are included in the article/supplementary material, further inquiries can be directed to the corresponding author.

## Ethics Statement

Written informed consent was obtained from the individual(s) for the publication of any potentially identifiable images or data included in this article.

## Author Contributions

JS and LS: performed the operation, collected information of patient, revised the literature, and drafted the manuscript. QF: revised the literature and the manuscript. All authors contributed to the article and approved the submitted version.

## Conflict of Interest

The authors declare that the research was conducted in the absence of any commercial or financial relationships that could be construed as a potential conflict of interest.

## Publisher's Note

All claims expressed in this article are solely those of the authors and do not necessarily represent those of their affiliated organizations, or those of the publisher, the editors and the reviewers. Any product that may be evaluated in this article, or claim that may be made by its manufacturer, is not guaranteed or endorsed by the publisher.
